# Sport activities for children and adolescents: the Position of the European Academy of Paediatrics and the European Confederation of Primary Care Paediatricians 2023—Part 1. Pre-participation physical evaluation in young athletes

**DOI:** 10.3389/fped.2023.1125958

**Published:** 2023-06-22

**Authors:** Anna Turska-Kmieć, Daniel Neunhaeuserer, Artur Mazur, Łukasz Dembiński, Stefano del Torso, Zachi Grossman, Shimon Barak, Adamos Hadjipanayis, Jarosław Peregud-Pogorzelski, Tomasz Kostka, Andrzej Bugajski, Gottfried Huss, Monika Kowalczyk-Domagała, Justyna Wyszyńska

**Affiliations:** ^1^ Department of Cardiology, Children’s Memorial Health Institute, Warsaw, Poland; ^2^ Polish Paediatric Society, Warsaw, Poland; ^3^ Working Group on Sports Cardiology, Polish Cardiac Society, Warsaw, Poland; ^4^ Sports and Exercise Medicine Division, University of Padova Department of Medicine, Padova, Italy; ^5^ Clinical Network of Sports and Exercise Medicine of the Veneto Region, Padova, Italy; ^6^ The European Academy of Paediatrics, EAP, Brussels, Belgium; ^7^ Institute of Medical Sciences, Medical College of Rzeszów University, Rzeszów, Poland; ^8^ Department of Paediatric Gastroenterology and Nutrition, Medical University of Warsaw, Warsaw, Poland; ^9^ Childcare Worldwide, Padova, Italy; ^10^ Adelson School of Medicine, Ariel University, Ariel, Israel; ^11^ Maccabi Health Services, Pediatric Clinic, Tel Aviv, Israel; ^12^ The European Confederation of Primary Care Paediatricians (ECPCP), Lyon, France; ^13^ Dana-Dwek Children’s Hospital, Tel Aviv Sourasky Medical Center, Tel Aviv, Israel; ^14^ School of Medicine, European University Cyprus, Nicosia, Cyprus; ^15^ Paediatric Department, Larnaca General Hospital, Larnaca, Cyprus; ^16^ Department of Paediatrics, Paediatric Oncology and Immunology, Pomeranian Medical University, Szczecin, Poland; ^17^ Department of Geriatrics, Medical University of Lodz, Lodz, Poland; ^18^ The Polish Society of Sports Medicine, Wroclaw, Poland; ^19^ Department of Physiotherapy, College of Physiotherapy, Wroclaw, Poland; ^20^ Kinder-Permanence Hospital Zollikerberg, Zollikerberg, Switzerland; ^21^ Working Group on Paediatric Cardiology, Polish Cardiac Society, Warsaw, Poland; ^22^ Institute of Health Sciences, Medical College of Rzeszów University, Rzeszów, Poland

**Keywords:** adolescents, children, physical evaluation, pre-participation, sport, young athlete

## Abstract

The European Academy of Paediatrics (EAP) and the European Confederation of Primary Care Paediatricians (ECPCP) emphasize the importance of promoting healthy lifestyles within the pediatric population. Many health professionals have questions concerning adequate levels of physical activity for both the healthy pediatric population and for those who may have specific complications. Unfortunately, the academic literature that provides recommendations for participation in sport activities within the pediatric population that have been published during the last decade in Europe is limited and is mainly dedicated to specific illnesses or advanced athletes and not toward the general population. The aim of part 1 of the EAP and ECPCP position statement is to assist healthcare professionals in implementing the best management strategies for a pre-participation evaluation (PPE) for participation in sports for individual children and adolescents. In the absence of a uniform protocol, it is necessary to respect physician autonomy for choosing and implementing the most appropriate and familiar PPE screening strategy and to discuss the decisions made with young athletes and their families. This first part of the Position Statement concerning Sport Activities for Children and Adolescents is dedicated to healthy young athletes.

## Introduction

The European Academy of Paediatrics (EAP) and the European Confederation of Primary Care Paediatricians (ECPCP) emphasize the importance of promoting healthy lifestyles for the pediatric population within every age range. The main conclusion from the study entitled “Youth sport participation trends in Europe - An output of Erasmus+,” which presented the findings of an international study on the involvement rates of youth in organized sports during 2017–2020, was a noticeable decline in participation in most sports for boys and girls (1). Therefore, there is an urgent need to increase support and retention of participation in sports from a young age along with essential physical activity (PA) counseling at every visit to the doctor.

Physical activity (PA) is essential for appropriate physical, emotional, and psychosocial growth of the pediatric population as well for patients with different diseases (2–4). The benefits of participation in sports are well-known in the scientific literature (2, 3, 5–8). The European Society of Cardiology (ESC) supports PA as a Class IA guideline for the prevention and management of cardiovascular (CV) disease (9). In 2022, the World Health Organization (WHO) suggested that children between the ages of 5 and 17 years accumulate a minimum of 60 min of moderate-to-vigorous PA (MVPA) per day that includes a variety of endurance activities (10). The WHO also recommends performing regular vigorous intensity aerobic activities and bone and muscle-strengthening activities at least 3 days a week for all age groups (5, 10, 11). Despite the widely accepted benefits of PA, recent data (2020) showed that the majority (81%) of children aged 11–17 years participate in MVPA less than 1 h a day with girls being less physically active than boys in most countries (12). Restrictions during the COVID-19 pandemic have affected children's PA levels. Therefore, promoting PA in the pediatric population should be a priority to recover pre-pandemic activity levels (13, 14).

## Aim

The sudden death of a young athlete (sudden athlete death, SAD) is a tragic and rare event, reported to occur in one to two cases out of 100,000 athletes each year (15, 16). A lack of knowledge of the current guidelines diminishes confidence in identifying children who may be at increased risk of injury (17–20) or even SAD (16, 21) as a result of participation in sports (22). Unfortunately, the academic literature that provides recommendations for participation in sport activities within the pediatric population that have been published during the last decade in Europe is limited and is mainly dedicated to specific illnesses or advanced athletes and not toward the general population. The aim of this EAP and ECPCP statement is not to present obligatory recommendations for sport pre-participation evaluation (PPE), but rather to summarize information based on analyses of the relevant literature and expert opinions regarding sports and exercise. This EAP statement reviews the key points of a patient's medical history and physical examination that should alert the clinician and guide healthcare professionals to make the best decisions for sports safety in their practice.

## Recommendations for physical activity and sport participation for the population of children and adolescents published in the literature

In 2019, the American Academy of Pediatrics (AAP) published “The Fifth Edition of PPE Monograph,” which is an approach to sports PPE that should be considered by healthcare professionals as a guide to help recognize whether a particular medical condition requires special care in young athletes (23).

In Europe, literature concerning sports PPE guidelines in the pediatric population is limited:

• In 2011, recommendations for PA, recreational sports, and exercise training for children and adolescents with congenital heart disease were published by the Exercise, Basic, and Translational Research Section of the European Association of Cardiovascular Prevention and Rehabilitation, the European Congenital Heart and Lung Exercise Group, and the Association for European Paediatric Cardiology (AEPC) (24).
• In 2012, the Polish Society of Sports Medicine (PSSM) published a position regarding age criteria during qualification for training in selected sport disciplines (25).
• In 2017, the EAP published a resolution related to health, integrity, and doping in sports for the pediatric population and young adults (26).
• Cardiovascular pre-participation screening recommendations for young athletes were published in 2017 by the AEPC (27).
• Guidelines for participation in competitive sports for patients with congenital heart disease (CHD) older than 16 years were published in 2020 by the Section of Sports Cardiology and Exercise of the European Association of Preventive Cardiology Group (EAPC) and the ESC working on adult CHD and AEPC (28). In 2020, the European Childhood Obesity Group and the EAP published specific birth to adolescence age-appropriate PA recommendations based on a search of the published literature (3).
• In 2021, the European Federation of Sports Medicine Associations (EFSMA) along with nine other organizations published “Pre-participation medical evaluation for elite athletes: EFSMA recommendations on standardized pre-participation evaluation form in European countries” (29).
• In 2021, the ESC published guidelines on the task force for cardiovascular disease prevention in clinical practice (30).

Age-specific pre-participation cardiovascular evaluation (PPCE) proposals for Swiss pediatric and adolescent athletes (under 18 years of age) were published in 2022 (31).

## Definitions

### Physical activity and sports

According to the WHO glossary of terms, PA is defined as “any active bodily movement resulting from the contraction of skeletal muscles that raises energy expenditure above resting metabolic level” (5). The types of PA included aerobic exercise, muscle strengthening, balance, and flexibility exercises (32) to be performed at least three times per week as part of minimum 60 min daily routine (33). The dose of exercise can be determined using the frequency, intensity, time, and type of exercise (FITT) principles (34). Detailed guidelines for following FITT principles for PA within the healthy pediatric population are described in literature (34, 35).

### Classification of sports and athletes

There are various types of sport activity which can be defined under different settings (5) such as:

• Leisure sports: recreational PA without pressure to play, continuous play, or play at a higher intensity based on the willingness of the subject. Any participants may stop or lower the intensity of their involvement at any time.
• Competitive sports: organized, competitive, and skillful PA within established rules of the sport.
• Exercise training: specialized, structured, planned methods and programs of PA used to enhance a participant's physical abilities.

A young athlete is defined as an individual, either amateur or professional, who participates in regular training and official sports competitions (36, 37). All PA can be described according to dynamic and static components, as shown in Table 1 (38).

**Table 1:** Classification of sports according to dynamic and static components (37).

Component		Increasing dynamic component in % of VO2max		
		A. Low (<50%)	B. Moderate (50%–75%)	C. High (>75%)
Increasing static component in % of MVC	I. Low (<30%)	Bobsledding/luge Field events (throwing) Gymnastics Martial arts Rock climbing Sailing Water skiing Windsurfing	Bodybuilding Downhill skiing Skateboarding Snow boarding Wrestling	Boxing Canoeing Kayaking Cycling Decathlon Rowing Speed skating Triathlon
	II. Moderate (10%–20%)	Archery Auto racing Diving Equestrian Motorcycling	American football Field events (jumping) Figure skating Rodeo Running (sprinting) Surfing Synchronized swimming “Ultra” racing	Basketball Ice hockey Cross-country skiing (skating technique) Lacrosse Running (middle distance) Swimming Team handball Tennis
	III. High (<10%)	Bowling Cricket Curling Golf Rifle Yoga	Baseball/softball Fencing Table tennis Volleyball	Badminton Cross-country skiing (classic technique) Field hockey Orienteering Race walking Racquetball/squash Running (long distance) Soccer

MVC, maximum voluntary contraction; VO2max, maximal oxygen uptake capacity or oxygen consumption.

The intensity of an endurance exercise program is described based on the percentage of a participant's VO2max. For muscle strength or resistance exercise, the intensity is classified as the ratio between muscle contraction and MVC.

Exercise trainings are specialized and planned methods and programs of PA for increasing functional capacity, performance, or cardiorespiratory fitness (39, 40). Recommendations should describe the amount (frequency, intensity, duration) and types of PA (4, 28, 34). Three stages of all exercise training are (1) a 10–15 min warm-up period, (2) the specific exercise training, and (3) a 10 min cool-down period with stretching. Adequate recovery time should be provided between training sessions (12–48 h depending on the intensity of the training session and conditioning of the participant).

## Age criteria for qualifying children and adolescents for participation in sports

### General age criteria for participation in sports

Pediatric and adolescent athletes who are younger than 18 years of age may be defined as participating in competitive sports at a high level if they are practicing in a team setting for at least 6 h per week (31). Not all exercises have the same consequences for an individual's health status, whether positive or negative (40, 41). The peak age range for involvement in sports is between the ages of 10–14 years, although currently athletes begin specialized training at an increasingly younger age (42). This may be due to greater pressure for achievement from coaches or parents (43).

In 2014, the United States Olympic Committee along with the National Governing Bodies used the long-term athlete development principles to create the American Development Model (42). They created five stages including the following: (1) discover, learn, and play (ages 0–12 years); (2) develop and challenge (ages 10–16 years); (3) train and compete (ages 13–19 years); (4) high performance or participate and succeed (ages ≥15 years); and (5) mentor and thrive (for life).

In 2012 in Europe, the Polish Society of Sports Medicine (PSSM) published its position regarding age criteria during qualification for training in selected disciplines in the pediatric population (25). The criteria were developed depending on developmental age, because calendar age is not always an accurate indicator of a child's growth (44). General age criteria for participation in sports proposed by the PSSM for children and adolescents are as follows (25): (1) younger than 6 years (all disciplines based on natural forms of movements in the form of game and play involving movement); (2) older than 6 years (the disciplines based on natural forms of movement, developing coordination of movement that does not exert a selective load on the skeletal system); (3) older than 8 years (sport disciplines that develop agility, speed, and movement dynamics); (4) older than 10 years (sport disciplines that develop endurance and strength); (5) older than 13 years (disciplines involving a full range of endurance training); and (6) older than 15 years (disciplines in which a full range of static strength exercises can be introduced).

Individual differences in growth and maturation can lead to unforeseen consequences in competitive sports and an increased risk of injury, especially in athletes who are constitutionally smaller and/or delayed in maturation. Therefore, there has been a long tradition of proposals for selecting athletes based on physical characteristics rather than chronological age. This strategy is called “bio-banding” and involves grouping and/or assessing athletes based on size and/or maturity status rather than chronological age (45). According to Tanner, biological age determined by puberty stages (46, 47) is more appropriate than chronological age and should be used in assessments of young athletes. Maturation is the process of a child's growth toward obtaining adult stature. All humans experience maturation differently. Females tend to mature faster than boys, but post-pubertal boys will experience greater increases in strength and power due to testosterone and other androgen hormones. Maturation should be measured in young athletes to properly monitor their growth and wellbeing (48). While bio-banding puts athletes into groups based on physical attributes, it does not preclude the inclusion of psychological and/or technical skills. For example, boys that may have matured early may be discouraged from competing or training with older participants if they lacked the technical competence and/or mental maturity to provide a safe and positive experience (49). Implications of somatic growth and maturation for sport participation, overuse injuries in adolescents during times of rapid growth, and concepts of bio-banding for both training and competition based on biological maturation for youth sports (50, 51) will be discussed in a separate part of the Position of the European Academy of Paediatrics and the European Confederation of Primary Care Paediatricians.

## Pre-participation physical evaluation

The main scope of sports PPE is to maximize safe participation. It should be individually adjusted to the participant especially during obligatory general pediatric consultations between the age of 0 and 18.

The objectives of the PPE are as follows (23, 52–56):

• Primary objectives: disclosure of defects that may limit participation and determine conditions that may be life-threatening or disabling.
• Secondary objectives: an indication of the general health of a child, examination of the level of maturation, provisions enabling an opportunity to answer questions, and introduction of the athlete into local sports.
• Other objectives: classification of an athlete according to individual qualifications, compliance with legal and insurance requirements for athletic programs, and provision of opportunities for children to compete.

In European countries, local and state requirements determine who has the authority to perform a PPE. General practitioners are responsible for conducting a PPE for children wishing to participate in sports, but in some countries, the determination of sport eligibility depends on the opinion of sport and exercise medicine (SEM) specialists, especially for top-level competitive sports (29, 57–62). The utility of the sports PPE in healthy populations has been questioned in recent years. The determination of relative contraindications should be made by an attending physician, who decides whether the patient is eligible for competitive sports or if further specific examinations are required. Although 3.2%–19.2% of athletes have a significant disclosure during the PPE, only 0.3%–1.3% of athletes are ultimately disqualified from involvement based on findings from the PPE (62–64). The Italian pre-participation evaluation program, diagnostic yield, rate of disqualification, and cost analysis, was published in 2020 (65). The PPE is commonly required by sports organizations to fulfill legal and insurance requirements for involvement in competitive sports, and mainly SEM specialists are qualified to conduct such PPEs (29).

The examination should be performed several weeks before a sporting event allowing time for additional examinations or treatment if needed. The AAP recommends a PPE at least 6 weeks prior to the beginning of the season to allow additional assessment and/or rehabilitation prior to competition (66). This may be too short of a time window because multiple specialists often collaborate with each performing a specific evaluation. The primary care physician needs to have time to assimilate all of the information for clinical decision-making and pre-competition screening.

Depending on governmental, state, or sport society requirements, a PPE should be repeated at least every 1–2 years or more frequently for selected sports or for selective health problems in athletes, especially in top-level athletes (59, 67, 68).

## Forms of pre-participation physical evaluation

The diagnostic components of PPE are health status, anthropometry, functional capacity, and exercise capacity. For the PPE, both the American and European Societies currently recommend a thorough investigation of personal history, family history, and a limited physical examination to determine the general health of the athlete (23, 29, 55, 59, 64, 66). The physical assessment should include vital signs such as visual acuity and cardiovascular, pulmonary, abdominal, skin, genitalia, neurologic, and musculoskeletal examinations. Parental verification of personal and family history is also recommended.

The EFSMA emphasizes the need for an implementation of a standardized PPE among European countries to ensure the best care for adult athletes (29). Until now, very few reports on periodic health evaluations by PPE in adolescent elite athletes exist (68–70). In 2009, the International Olympic Committee published a Consensus Statement on the Periodic Health Evaluation of Olympic athletes, explaining the purposes and procedures of the PPE in adults (68). The youth PPE was shown to be efficient in diagnosing different disorders, enabling rapid treatment in 12% of Italian Olympic athletes (69). The AAP published standardized physical examination forms as tables with questions about family and athlete history and physical exams, as well as for young athletes with special needs for clearance decisions concerning participation in sports (23).

The pre-participation evaluation does not only focus on elements that determine known problems that affect sport involvement (71). During a PPE, a consulting physician can also educate the participants about injury prevention or concussion, first-aid and external defibrillator–cardioverter support performance, dietary counseling (72), and the effects of nutritional supplements (73). Finally, the PPE may provide an appropriate approach for preventing any doping practices (26).

The past several years, especially during the COVID-19 pandemic, has seen a rapid growth of electronic sports (eSports) media, thanks to the increasing availability of online games and broadcasting technologies (74), and numerous injuries may occur in the participants (eAthletes) (75). The potential benefits of a pre-participation evaluation and injury prevention program in eSports will be discussed in a separate part of the Position of the European Academy of Paediatrics and the European Confederation of Primary Care Paediatricians.

## General considerations during a PPE to assess the overall health of a young athlete

wIt has been reported that in up to 58% of young athletes who do not positively pass the PPE screening, the decision to deny may be based on medical history alone or dizziness with exercise, history of asthma, body mass index (BMI), elevated systolic blood pressure, visual acuity, heart murmur, or problems detected during musculoskeletal examination (76). A pre-participation health evaluation in a cohort of adolescent athletes competing in Youth Olympic Games showed that PPE led to a diagnosis of pathological conditions in 12% of the population (69). The most prevalent diseases identified were cardiovascular and pulmonary diseases (4.5% each), followed by endocrine alterations (2%) and infectious, neurological, and psychiatric disorders (0.4% each). Cardiovascular disease was found in 3.9% of elite adult athlete cohort participants who underwent a comprehensive cardiovascular evaluation prior to participation in the Olympic Games (77).

### Personal history

Personal history should identify past red flags and present medical conditions as well as previous surgeries and associated consequences such as past injuries, history of passing out, episodes of syncope or dizziness, any medicines currently being taken, vaccinations, history of allergies and anaphylaxis, infection diseases, headache, seizures, and any potential cardiovascular warning signs (high blood pressure, heart murmur, high cholesterol, myocarditis) (23, 56). Boys should be diagnosed for testicular pain. Girls should be asked about their menstruation. The physician should also inquire about smoking and vaping, alcohol intake, drugs, diet pills, or supplements, including steroids.

It is very important during a PPE to inquire about the actual type, amount, and intensity of exercise training, associated chest pain or heart palpitations experienced during or post exercise, trouble breathing during exercise, dizziness or syncope during exercise, and the presence of excessive, unexpected, or unexplained fatigue during exercise.

During a PPE, the consulting physician should inquire about dietary habits and eating disorders. Suggested diets for young athletes were presented in a Position Statement of the American Dietetic Association, Dietitians of Canada, and the American College of Sports Medicine (72). Further important components of a PPE are screening for mental health disorders (78). A list of recommended personal history questions from different systems during PPE in young athletes are summarized in Table 2.

**Table 2:** List of recommended personal history questions from different systems during sports PPE in young athletes.

System	Question	Sport qualification
Circulatory problems	Syncope during rest—vasovagal	Possible sport qualification without cardiologist consultations
	Syncope during rest—other than vasovagal	Need cardiologist consultation before sport qualification
	Syncope during or after exercise	
	Arrhythmia (irregular rhythm, palpitations)	
	Dizziness during fast and/or irregular rhythm	
	Lower physical efficiency than contemporary children after severe infection	
	Chest pain during or after exercise	
Infection problems	History of recent viral infection with cumulative fatigue and myocarditis suspicion	Cardiologist consultation before sport qualification ought to be considered before sport qualification
	History of Kawasaki disease with cardiac involvement	
	Post-COVID infection with cardiac involvement especially post-PIMS	
	HIV, HBV, HCV	Athlete education is recommended to limit pathogen transmission addressed both to universal precautions and to avoid high-risk behaviors
	History of infectious mononucleosis	After consultation with primary care physician return to non-contact light sport activities for 3–4 weeks after the onset of symptoms of infection mainly to reduce the risk of splenic rupture
Nervous system problems	History of concussion or unexplained fainting, seizures, or near drowning	Need neurologist consultation before sport qualification
	History of head injury that caused concussion	
	History of prolonged headaches especially with exercise	
	History of numbness, had tingling, weakness in arms or legs	
	History or risk factors for exertional stroke	
Eye disorders and vision problems	History of any eye injury/surgery, vision disorders, wear glasses/contact lenses	Need ophthalmologist consultation before sport qualification
Laryngological problems	History of hearing loss	Need laryngologist (ENT) consultation before sport qualification
Pulmonary problems	Dyspnea, lower physical efficiency than contemporary children, exercise-induced asthma	Need pulmonologist and/or cardiologist consultation before sport qualification
	Dyspnea or cough during usually the first 20 min of effort or 5–10 min after exercise	Suspected exercise-induced asthma—need pulmonologist consultation before sport qualification
	Controlled asthma	Possible sport qualification
	Pharmacologically non-controlled frequent asthma episodes	Sport activities are contraindicated. Need pulmonologist consultation
	Recurrent bronchitis in early childhood without actual episodes of dyspnea or couch during or after exercise	Possible sport qualification without pulmonologist consultations
	Asthma only in family members	
Endocrinological problems	History of diabetes mellitus type 1 or 2, use of insulin pumps	Need endocrinological consultation before sport qualification
Gastrointestinal system	Inflammatory bowel disease	Sport activities are contraindicated during period of exacerbations, moderate degree of sport activities is allowed during remission period after gastroenterologist consultation. History of chronic diarrhea Greater risk of dehydration
Genitourinary problems	Nephrotic syndrome	Sport activities are contraindicated during the period of exacerbations, moderate degree of sport activities is allowed during remission period after nephrologist consultation
	Post-exercise proteinuria	Possible sport qualification only if sporadically observed, if more intensive and frequent need nephrologist consultation
	Single kidney, single gland	Trauma sports are contraindicated
	Menstrual disorders	If female athlete triad is suspected, sport activities are contraindicated without consultation of a gynecologist
Osteoarticular problems	Knee pain	Need orthopedist consultation before sport qualification
	Arthralgia in other localizations	Need orthopedist or rheumatologist consultation before sport qualification
	Broken bones, joint dislocation, or twisting in medical history without actual symptoms	Possible sport qualification without orthopedist consultations
Dermatologic problems	History of skin infection	Temporary restriction in sport participation
Mental problems	History of depression, anxiety disorders, attention deficit/hyperactivity disorder	These athletes may produce a variety of psychological responses negatively affecting sport participation. May need comprehensive mental healthcare. Screening for mental health disorders is an important component of PPE

PPE, pre-participation evaluation; PIMS, pediatric inflammatory multisystem syndrome; HBV, hepatitis B virus; HCV, hepatitis C virus.

### Family history

The PPE family history is designed to recognize athletes at risk, especially in those with a family history including SAD. Most sudden deaths have been attributed to congenital or acquired cardiovascular malformations (36, 79). Other causes of sudden death include heat stroke, cerebral aneurysm, asthma, commotio cordis, and sickle cell trait (80). Any athlete with a family history of the following conditions requires a full evaluation and cardiological follow-up (23, 24, 28, 29, 59, 81, 82). If anyone in the athlete's family:

• Died of heart problems or had sudden cardiac arrest before the age of 50 years or died for no apparent reason before the age of 50 years [to include sudden infant death syndrome (SIDS), unexplained car accident, drowning].
• Had any type of heart problem, primary pulmonary hypertension, arterial hypertension, family and hypercholesterolemia.
• Had syncope, presyncope, or unexplained seizures.
• Had significant arrhythmia, an ablation procedure, an implanted pacemaker, or defibrillator.
• Had exercise-induced asthma.
• Had any of the following genetic disorders including hypertrophic cardiomyopathy (HCM), dilated cardiomyopathy (DCM), arrhythmogenic right ventricular cardiomyopathy (ARVC), Marfan syndrome, and Ehlers–Danlos syndrome and channelopathies such as Brugada syndrome (BRS), long-QT syndrome (LQTS), or short-QT syndrome (SQTS).

Sudden death in young athletes is rare and is usually caused by a genetic or congenital structural cardiac disorder (83–85), so detailed family history may help in identifying athletes especially at risk of primary arrhythmia syndromes (23, 27, 67). The issues raised within a family history may be quite obscure to the athlete and family, but young athletes and their parents should still be informed by their physician why such data from the PPE are essential for lowering SAD risk. Importantly, any abnormal finding in the family history and/or physical examination including abnormal prior cardiovascular testing results and a positive family history including premature death (<50 years) and/or genetic cardiomyopathy in a first-degree relative requires further investigation, as suggested by international guidelines (15, 36).

## Significant elements of a physical examination

Physical examinations must primarily screen the musculoskeletal, cardiovascular, and nervous systems. Abnormal breath sounds in the lungs should be diagnosed. The PPE may also include a dental and vision evaluation (risk of soft tissue lesions). The evaluation of vision can be performed using a standard vision chart (standard Snellen eye chart) with specification on the use of glasses or contact lenses.

A critical component of a PPE is an anthropometric examination that includes somatoscopy, taking into account measurement of weight, height, BMI, sitting height, body composition, and girth measurements (29, 85, 86). Table 3 contains a summary of significant elements of a physical examination from different systems during a sports PPE.

**Table 3:** List of significant elements of physical examination during sports PPE.

Body layout or area	Elements of physical examination	Comments
Circulation	Heart rate	Bradycardia may be present in trained athletes, and no further evaluation is needed unless accompanied by fainting or unconsciousness. Resting tachycardia requires further diagnosis and multiple checks; in the case of persistent tachycardia, 24 h Holter ECG monitoring and cardiological consultation; not make a judgment pending clarification. Other cardiac arrhythmias: 24 h Holter ECG monitoring and cardiac consultation; not make a judgment pending clarification
	Blood pressure	Assessment on percentile grids, in the case of hypertension (≥95th percentile for age, sex, and height), do not issue a judgment until effective correction is made, refer for a consultation to a pediatric cardiologist, nephrologist, or hypertensiologist
	Heart rate symmetry in the extremities	In case of asymmetry, refer to a cardiologist and for echocardiography, judgment pending on clarification
	Heart murmur	Issue a judgment if the criteria for an innocent murmur are met (note: a mild murmur may occur in heart defects with very little hemodynamic disturbances); if the criteria are not met, do not issue a judgment until clarification, and refer the patient to a cardiologist and for echocardiography
	Cyanosis	In the case of central cyanosis, refer to a cardiologist and for echocardiography, judgment pending on clarification
Respiratory	Shortness of breath, wheezing, or other auscultation phenomena	If the symptoms are related to an acute infection, the patient should be reassessed after recovery. If symptoms persist, the patient should be referred to a pulmonologist and spirometry Judgment pending on clarification
	Nasal obstruction	ENT consultation is recommended for swimming or diving people, no contraindications for practicing other sports
Abdominal cavity	Enlarged liver or spleen	Contraindicated contact sports, cycling, and climbing
Oral cavity	Tooth pain	In the case of caries, a dental consultation is recommended
Osteoarticular	Flat feet	If it is severe, consult an orthopedist; the decision may be issued if practicing sports does not cause pain
	Valgus in the ankles	
	Valgus in the knee joints	
	Unequal length of the lower limbs	
	Mobility in the hip joints	If there is a visible restriction of mobility, you should refer to an orthopedist; if practicing sports does not cause pain, you can issue a decision
	Achilles tendon contracture	If it is severe, consult your orthopedist before making a judgment
	Pain in the attachment of the Achilles tendon to the calcaneus under pressure	If severe, do not issue a judgment until an orthopedic opinion has been obtained
	Soreness of the calcaneus with pressure	If severe, do not issue a judgment until an orthopedic opinion has been obtained
	Soreness or thickening of the tibial tuberosity under pressure	If severe, do not issue a judgment until an orthopedic opinion has been obtained
	Instability in the knee joint	
	Pain with pressure on the kneecap	
	Meniscal tests	If the result is positive, an orthopedic consultation is indicated If practicing sports does not cause pain, a judgment can be made
	Flabbiness of the joints	If it is severe, genetic consultation to assess collagenopathy is indicated (most often Ehlers–Danlos syndrome). If there are no recurrent dislocations or sprains of the joints, a decision can be made
	Asymmetry of the shoulders and shoulder blades	Judgment can be made
	The presence of correct spine curvatures	If a significant enhancement or elimination of the correct curvatures of the spine, consultation with a medical rehabilitation physician or orthopedist is recommended. If practicing sports does not cause pain, a decision can be issued
	The presence of scoliosis	In the case of significant scoliosis, the decision to allow competitive sports depends on several variables, including the degree of load on the spine and the degree of curvature of the spine Some doctors make the consent to practice a specific sport of a certain intensity dependent on the presence of pain in the spine. Training can be continued by a patient with scoliosis who does not develop back pain after training
	Waist asymmetry	Assessment of the length of the lower limbs and scoliosis; if it is severe, an orthopedic consultation is necessary. If practicing sports does not cause back pain, a judgment can be made
	Restriction of mobility in the shoulder joints	If it is severe, an orthopedic consultation is necessary
	Examination of other joints depending on previous injuries and reported ailments	If in doubt, consult an orthopedist

PPE, pre-participation evaluation.

### Musculoskeletal system

Evidence for injury incidence and the long-term effects of participating in sports in the pediatric population are limited (17, 18, 87, 88). A musculoskeletal assessment is recommended to examine joints (89, 90), physical function (flexibility, posture, gait, muscle strength, joint laxity), and regional abnormalities (spine, upper extremity, lower extremity) (55, 62, 91). Of the conditions identified during the PPE, 14% required follow-up before clearance for participation (92, 93).

The first component of the orthopedic PPE is a complete history of previous injuries and surgeries. A previous musculoskeletal injury is a major risk factor for re-injury, especially if the original injury was not rehabilitated properly (92, 94).

The second component of the orthopedic PPE is the physical examination. The examination should evaluate spinal anomalies, deformity, and back pain (95). Back pain was reported in about 8% of young teenage athletes mainly from combat sports, team sports, explosive strength sports, and endurance sports (96). It is known that injury in anatomic regions such as the knees, ankles, and shoulders have a higher risk of future injury (97).

Suggested examples of quick musculoskeletal examinations are offered by the American Medical Society for Sports Medicine (AMSSM) in online educational resources (98). In 2021, the American College of Sports Medicine published Musculoskeletal and Sports Medicine Curriculum Guidelines for Pediatric Residents (99).

### Nervous system

The neurological assessment is focused on the abnormal conditions observed during present and previous examinations (100, 101). Youth with a history of concussion should have a neurological assessment (102). Vision and/or hearing loss must be corrected, and consultation with an ophthalmologist or laryngologist may be necessary.

### Abdomen

An abdominal assessment can be brief in the absence of a significant history of gastrointestinal (103–106) or nephrological diseases (81, 107–109). Splenic enlargement and history of recent infectious mononucleosis (Epstein–Barr virus infection) are temporary contraindications to sports because splenic rupture is a risk (110). An examination for boys should include assessment of the groin and genitalia. Privacy and chaperones should be present when such evaluations are included, asking for their consent (a second healthcare professional should stay in the room during these examinations). Genitourinary assessment in boys is particularly justified in the case of collision sports, because it is necessary to further protect boys having a single testicle.

### Cardiovascular system

Most of the cases of SAD occur in individuals with a pre-existing cardiac abnormality, and in young athletes (<35 years), instances of SAD are mostly due to inherited or congenital cardiac disorders. In 2005, the Study Group of Sports Cardiology of the Working Group on Cardiac Rehabilitation and Exercise Physiology and the Working Group on Myocardial and Pericardial Diseases of the ESC published a common European protocol for cardiovascular pre-participation screening of young competitive athletes for the prevention of SAD which are termed “the Lausanne recommendations” (111). A position paper from 2017 promoted by the EHRA, and the EACPR reviewed the evidence regarding the appropriate diagnostic methods to determine selected heart conditions at risk in SAD (15).

It is obligatory during a PPE to inquire about cardiovascular disease in family history (112, 113) with a high life-threatening risk of cardiac arrest during exercise in young athlete (114). Since 1982, it has been mandatory for every Italian competitive athlete to undergo an annual PPE that includes an assessment of the CV system including non-CV evaluations to identify diseases that pose a risk of SAD during sports or other conditions that may threaten the athlete's health (113, 115). The ESC (111) and the AHA (116) suggested the adoption of a 14-element SAD screening tool during the PPE that contains medical history questions and four assessment elements. Table 4 presents a proposed set of screening questions concerning cardiological problems to be addressed during a PPE.

**Table 4:** The 14-element ESC (92) and AHA (96) questionnaire for pre-participation cardiovascular screening of competitive athletes.

Personal history	1. Chest pain, discomfort, tightness, pressure related to exertion 2. Unexplained syncope or near-syncope judged not to be of neurocardiogenic (vasovagal) origin; of particular concern when occurring during or after physical exertion 3. Excessive and unexplained dyspnea/fatigue or palpitations, associated with exercise 4. Prior recognition of a heart murmur 5. Elevated systemic blood pressure 6. Prior restriction from participation in sports 7. Prior testing for the heart, ordered by a physician
Family history	8. Premature death (sudden and unexpected or otherwise) before 50 years of age attributable to heart disease in one or more relatives 9. Disability from heart disease in a close relative younger than 50 years 10. Hypertrophic or dilated cardiomyopathy, long-QT syndrome, or other ion channelopathies, Marfan syndrome, or clinically significant arrhythmias, specific knowledge of genetic cardiac conditions in family members
Physical examinations	11. Heart murmur refers to heart murmurs judged likely to be organic and unlikely to be innocent; auscultation should be performed with the patient in both the supine and standing positions (or with Valsalva maneuver), specifically to identify murmurs of dynamic left ventricular outflow tract obstruction 12. Femoral pulses to exclude aortic coarctation 13. Physical stigmata of Marfan syndrome 14. Brachial artery blood pressure (sitting position)—preferably taken in both arms

The use of an electrocardiogram (ECG) is proposed as a screening tool in young athletes to identify potentially life-threatening arrhythmias (e.g., channelopathies, pre-excitation syndromes), structural congenital heart disease, and cardiomyopathies (21, 111). Data relating to the use of ECG during a PPE are controversial especially in the United States (117, 118) but may be required for highly trained athletes by some sports organization. In the absence of clear evidence, the AMSSM respects physician autonomy in implementing an ECG as the most appropriate PPE cardiovascular screening strategy (117). The EFSMA statement from 2015 on ECG for PPE concluded that ECG is very sensitive in heart screening (119). In Italy, cardiovascular PPE protocols including ECG prior to competitive sports practice was adopted by the ESC in 2005 as the “common European protocol” (111) and implemented by many National European Sport Associations during screening of top-level adult athletes (120, 121). In Europe, cardiovascular screening that includes ECG before playing sports in youth is supported by some governing bodies. In selected individual athletes, further non-invasive or invasive cardiac evaluation may be required before the final decision of clearance for participation after the initial cardiovascular PPE (111, 116). In Switzerland, it is recommended that all young athletes practicing with a team with a training load of at least 6 h per week undergo PPCE based on medical history and physical examination from the age of 12 years onward. Inclusion of a standard 12-lead ECG evaluation is also suggested for all post-pubertal athletes (or older than 15 years) with an analysis in accordance with the International Criteria for ECG Interpretation in Athletes (31).

## Specific individual history and physical examination forms

### Laboratory tests

There are no routine laboratory tests that have been proven to be useful and cost-effective as elements of a PPE (67). Routine laboratory or other tests during the PPE have not been supported by sports medicine societies (67, 122).

### The female and male athlete triad syndrome in top-level young athletes

The female athlete triad syndrome with symptoms of amenorrhea, decreased bone mineral density (frequent stress fractures, osteoporosis), and disordered eating may appear in the absence of a balanced diet (123–125). The male athlete triad syndrome occurs most frequently in adolescent and young adult male endurance and weight-class athletes and includes low energy availability with or without disordered eating, functional hypothalamic hypogonadism, and osteoporosis or low bone mineral density (125, 126). In 2014, the International Olympic Committee (IOC) changed the term athlete triad to relative energy deficiency in sports (RED-S) (127). This concept allows identification of energy deficiency as a key to the disruption of several physiological functions of different areas, such as reproduction, bone, endocrine, metabolic, hematological, growth and development, physiological, cardiovascular, gastrointestinal, and immunological, with consequences for the performance and health of the athlete in general (128).

### Doping laws

During a PPE, it is necessary to inform a child or adolescent who is eligible for competitive sports that he or she must meet the requirements of doping laws (26, 129, 130). Establishing a Therapeutic Use Exemptions Certificate is therefore mandatory if subjects need to take any of the prohibited substances or medical therapies listed each year by the World Anti-Doping Agency (www.wada-ama.org) (131).

### Sport disciplines associated with increased risk of undesirable health consequences

Sports recommendations for young athletes should also be concerned with the particular form of PA. Healthcare providers should recognize individual risks for the pediatric athlete in participating in the sport discipline chosen, because some sport disciplines may have particular constraints and may be subject to specific medical examinations. According to the government regulations in France, sport disciplines with a higher risk of injury for athletes are as follows (130, 132): (1) practiced in a specific environment (mountaineering, underwater diving, caving), (2) competition fighting, in which the fight may end as a result rendering them unconscious and incapable of defending themselves, (3) involving the use of firearms or compressed air, (4) involving the use of motorized land vehicles (except for radio-controlled automobile model making), (5) aeronautical sport disciplines practiced in competition (except for model aircraft), (6) parachuting, and (7) rugby fifteens, rugby thirteen, and rugby sevens.

Some PA might be related to a higher risk of injury if unconsciousness occurs (e.g., in swimming, climbing, or horseback riding), and some other types of PA have a high risk of collision or trauma (e.g., in athletes with implanted electrical cardiological devices or with congenital bleeding disorders who are taking anticoagulants) (19, 24, 28, 133). Sport disciplines have been classified as non-contact, limited contact, or with contact/collision which were described in detail by the AAP (23, 52).

The safe age categories for combat sport disciplines are proposed below with criteria that are considered in the most frequently practiced sport disciplines: (1) participation in boxing for children and youth is not recommend, and (2) children younger than 12 years may participate only in non-contact sports involving training and competition in a form of technique demonstration or directed combat with task performance. They can participate in competition with non-contact activities. In disciplines not involving kicking or hitting, children can participate in training and competition from the age of 9–10 years in accordance with the regulations of sport federations, and (3) youth older than 12 years, who have participated in training and non-contact disciplines for at least 9 months, may participate in classes and training sessions and compete in combat sports with limited contact if the training or competition does not involve fighting using hand or leg blows with full force (25).

In all combat sports that include hitting or kicking, or use of weapons, participants should use protection for the mouth, genitals, breast (girls), and other body parts according to regulations (25). Table 5 contains a list of recommended significant personal history questions to be asked during a PPE before qualification for selected sport disciplines for young athletes associated with increased risk of undesirable health consequences.

**Table 5:** List of recommended diagnostic examinations during the first pre-participation physical evaluation and repeated every 1–2 years during repetitive control evaluation of young athletes.

Type of examination	PPE	Control PPE
		Every 1–2 years
Obligatory examinations		
Family and personal history	Yes	Yes
Physical examinations	Yes	Yes
The condition of the dentition	Yes	Yes
Visual screening (standard Snellen eye chart)	Yes	Yes
12-lead electrocardiogram	Yes	Yes
Orthopedic evaluation	Yes	Yes
Optional examinations for selected athletes		
Laboratory tests	Depending on the results of PPE	
Spirometry	Scuba diver	
Neurological consultation	Combat sports	

PPE, pre-participation evaluation.

## Young athlete medical eligibility form

After the results of the PPE are analyzed, athletes will likely be classified according to one of the following (29):

• Medically eligible for sports performance without restriction.
• Medically eligible with recommendation for further management.
• Not medically eligible for sports performance: for all sport disciplines or a selected discipline, temporary or constantly contraindicated.

Healthcare professionals should provide written guidelines with restrictions and permissions regarding adequate levels of PA and exercise. Determination of sports eligibility depends on historical and current examination findings and the sport in which the athlete desires to participate. Fever, acute illness, and viral myocarditis are conditions temporarily limiting sport participation. Limiting activity is also important for preventing complications such as dehydration or thermoregulatory problems. Some athletes will require further re-evaluation (e.g., referral to cardiology, neurology, or orthopedic specialists) (28, 95, 99, 100) and treatment (e.g., treatment of hypertension or exercise-induced bronchospasm, ablation of accessory pathway in WPW syndrome, implantation of cardioverter defibrillator) (28), or they should be included in a physiotherapy program (e.g., after an orthopedic operation) before a return to play (RTP). Participation in PA should be assessed regularly.

Table 6 contains a list of proposed diagnostic examinations during the first PPE which should be repeated every 1–2 years for a repetitive control evaluation of young athletes.

**Table 6:** List of recommended significant personal history questions during PPE before qualification to selected sport discipline in young athletes with increased risk of undesirable health consequences.

Sport discipline	Diseases	Questions	Comments
Swimming, diving	Otolaryngologist (ENT)	Otitis media	If it recurs, consult an ENT specialist, and do not issue a judgment until clarified
		Otitis externa	
		Perforation of the tympanic membrane	Swimming and diving are contraindicated until the eardrum is atrophied
		Chronic otitis media with effusion	The attending ENT specialist should be consulted before making a judgment
		Placement of tympanostomy tubes	Contraindication to diving, in the case of swimming, consult the attending ENT specialist before issuing the ruling
		Recurrent sinusitis of the nose	The attending ENT specialist should be consulted before making a judgment
		Curve of the septum of the nose	The attending ENT specialist should be consulted before making a judgment
		Chronic runny nose	
	Cardiological	Syncope/palpitations during exercise, family history of syncope, or sudden cardiac death	Contraindication to swimming in selected channelopathies (long-QT syndrome), electrocardiogram should be performed before making a judgment
Sailing, canoeing, rowing, windsurfing, and other water sports (kitesurfing, wakeboarding, etc.)	ENT	Perforation of the tympanic membrane	Until atresia of the tympanic membrane is contraindicated
		Placement of tympanostomy tubes	No contraindications
	Neurological	Epilepsy	Contraindicated
Judo	Orthopedic	Cervical spine pain	Perform functional x-ray of the cervical spine; if in doubt, consult an orthopedist
		Cervical spine instability	Not make a judgment pending clarification
		Dizziness	Contraindicated
Martial arts	Neurological	Epilepsy	A neurological consultation and a functional x-ray of the cervical spine are recommended to assess the stability
		Dizziness	Not make a judgment pending clarification
		Defective focal symptoms	Contraindicated, neurological consultation and functional x-ray of the cervical spine are recommended to assess the stability Not make a judgment pending clarification
		Loss of consciousness	
		Loss of consciousness during a fight after a blow to the head (known as a knockout)	
	Dental	Braces	Dentist consultation

PPE, pre-participation evaluation.

## Evaluation for resumption of physical activity/sport activity for a child/adolescent with COVID-19 infection

Serious consideration needs to be given to RTP policies regarding COVID-19 infections in the pediatric and adolescent population. The AAP guidance from 2022 suggested that all eligible participants should receive a primary course of COVID-19 vaccine or booster doses when recommended (135). A history of COVID-19 infection and symptoms should also be included in the PPE (135). Any young athlete who tests positive for current COVID-19 infection, even if asymptomatic, should avoid all training and games until cleared by a physician. Long COVID is diagnosed when symptoms persist for more than 3 months (136). This condition includes many clinical symptoms such as fatigue, breathlessness, brain fog, depression, and inability to return to normal PA. Coronavirus disease has also created a new condition termed pediatric multisystem inflammatory syndrome (PIMS) (137). Multi-organ changes during PIMS can involve the cardiovascular, respiratory, kidney, gastrointestinal, and neurological systems.

The decision to return to PA after COVID-19 should take into account the duration of the disease, the severity of respiratory or cardiac symptoms (138), as well as the presence of comorbidities, and the capacity and the intensity of the planned exercise (139). In 2022, the Scientific Council of the Deutsche Gesellschaft fur Sportmedizin und Pravention (DGSP) published guidelines for returning to sports after COVID-19 that was addressed to elite athletes (140). It recommended limiting PA with a 3-day training pause after the time of diagnosis for an asymptomatic course of COVID-19. Mild competitions may be possible after a total of 10 days have elapsed after the symptoms have disappeared. In a recent document from 2022, the AAP recommends that members of the pediatric population have a minimum of 2-week rest period without exercises (134). Symptomatic patients should limit PA for 2–4 weeks. In Europe, athletes are advised to wait to RTP for a minimum of 10 days from the onset of symptoms, plus a minimum of 7 days from the resolution of COVID-19 symptoms (141). Because of the limited information on COVID-19 and exercise, it is strongly advised that all athletes who have had COVID-19 be cleared for participation by their primary care physician (134). If primary care physicians have any questions regarding their patients’ readiness to return to competition, they should feel free to refer them to a pediatric medical subspecialist. Any child with a history of a positive COVID-19 test, regardless of whether they had symptoms, should be screened for chest pain, shortness of breath, syncope, and palpitations and have a complete physical exam (142, 143). Youth who have had moderate or severe symptoms of COVID-19 should be referred to a cardiologist (144, 145).

Table 7 summarizes sports recommendations based on the severity of symptoms for children and adolescents according to the WHO definitions of COVID-19 infection (146).

**Table 7:** Recommendations for resumption of physical activity/sport activity based on the severity of symptoms for children and adolescents with COVID-19 infection.

COVID-19 infection symptom severity	Recommendations	Follow-up procedures
Asymptomatic children or adolescents with positive COVID-19 test or mild symptoms managed at home: <4 days of fever >38°C; <1 week of myalgia, chills, and lethargy)	Follow-up video visit, phone call, or other electronic communication (e.g., portal message) with primary care physician is recommended. Individuals who are asymptomatic or have mild symptoms who complete their 5-day isolation should be fever-free off all fever-reducing medication and have improving symptoms for a minimum of 1 day prior to beginning a return to PA progression. It is recommended that these children and adolescents update their pediatrician’s office via a phone call to ensure the history of COVID-19 infection is added to their medical record	For children and adolescents with a history of COVID-19 infection** **who have already advanced back to PA/sports on their own and do not have any abnormal signs/symptoms, no further workup is necessary. All athletes and their parents should be provided with guidance to monitor for signs/symptoms concerning myocarditis as they return to PA. This includes monitoring for any onset of chest pain, shortness of breath out of proportion for upper respiratory tract infection, new-onset palpitations, or syncope. These symptoms are indications for stopping PA and seeking immediate medical care; consultation with a pediatric cardiologist should be encouraged
Moderate symptoms of COVID-19: ≥4 days of fever >38°C), ≥1 week of myalgia, chills, or lethargy, or a non-ICU hospital stay and no evidence of PIMS	An evaluation by their PCP is recommended, as these patients may be at greater risk for subsequent cardiovascular disease Athletes who test positive for COVID-19 should not exercise until they are cleared by a physician Primary care physician evaluation is currently recommended after symptom resolution and completion of isolation. The PCP will review the American Heart Association 14-element screening evaluation with special emphasis on cardiac symptoms including** **chest pain, shortness of breath out of proportion for upper respiratory tract infection, new-onset palpitations, or syncope** **and perform a complete physical examination and an ECG	If cardiac workup is negative, gradual return to PA may be initiated after 10 days have passed from the date of the positive test result, and a minimum of 1 day of symptom resolution (excluding loss of taste/smell) has occurred off fever-reducing medicine. If cardiac sign/symptom screening is positive or ECG is abnormal, referral to a cardiologist is recommended. The cardiologist may consider ordering a troponin test and an echocardiogram at the time of acute infection. Depending on the patient’s symptoms and their duration, additional testing including a Holter monitor, exercise stress testing, or cardiac magnetic resonance imaging may be considered. It is recommended that these athletes must be cleared to resume participation by their pediatrician and/or pediatric medical subspecialist, who provides care for that particular system
	Athletes may have a persistent cough for 4 weeks after a COVID-19 infection. Moderate to severe pneumonia can also have severe impacts on the pulmonary vasculature, so any new-onset breathlessness or chest pain should include embolism on the differential and be tested	If there are worsening symptoms or new symptoms (new productive cough, chest pain, dyspnea), resumption of PA/sport activity should cease, and reassessment should rule out pneumonia, embolism, or post-inflammatory bronchoconstriction. Testing should include chest x-ray, ECG, lung function tests, and biomarkers for inflammation, myocyte necrosis, and thromboembolic disease (CRP, troponins, and d-dimer tests). If the underlying cause is still unclear, escalate to computed thorax tomography (if there are concerns for thromboembolic pathology) and cardiopulmonary exercise testing. It is recommended that these athletes must be cleared to resume participation by their pediatrician and/or pediatric medical subspecialist. who provides care for that particular system
Severe COVID-19 symptoms (e.g., hypotension, arrhythmias), requiring support ICU stay with intubation and/or ECMO or PIMS	Children and adolescents with diagnosis of myocarditis or PIMS with cardiac involvement. Restricted from exercise for a minimum of 3–6 months and obtain cardiology clearance prior to resuming training or competition	Other testing may be ordered based on the child or adolescent’s signs and symptoms. Coordination of follow-up cardiology care should be arranged prior to hospital discharge. It is recommended that these athletes must be cleared to resume participation by their pediatrician and/or pediatric medical subspecialist who provides care for that particular system
Resumption of PA/sport activity (AAP recommendations) for children and adolescents	All children younger than 12 years	Progress back to sports/physical education classes according to their own tolerance once the above steps for isolation and clearance have been completed
	Individuals who are 12 years and older perform the following progression once isolation is completed and physician clearance has been obtained (if indicated): Notice: A face mask should be worn for all PA, including games or scrimmages, until 10 full days from positive test or symptom onset have passed	Minimum 1 day symptom-free (excluding loss of taste/smell), tolerating ADLs Also prior to return to games is recommended for individuals asymptomatic or with mild symptoms: 1 day of practice; for individuals with moderate symptoms, one light practice or 30 min minimum of cardiovascular exercise, and 1 day full practice

PIMS, pediatric multisystem inflammatory syndrome; ECMO, extracorporeal membrane oxygenation; AAP, American Academy of Pediatrics; PA, physical activity; PCP, primary care physician; ADLs, activities of daily living.

When returning to PA, children and adolescents should gradually increase the frequency, training volume (duration of training session, miles, repetitions), and intensity of activity to avoid injury. Based on an assessment of current and previous activity levels, young athletes should return to activity at 25%–50% of the volume and intensity at which they were participating previously (147). Finally, it is important to consider not only the physical but also the mental wellbeing of athletes before RTP (148). Counseling or psychotherapy should be considered for those having difficulty coping or for those experiencing emotional distress (149).

## Sports and exercise medicine

Sport and exercise medicine is a new multidisciplinary medical specialty that supports all subjects and patients who want to engage in PA, exercise, and sports. These medical specialists are also experts regarding the performance of athletes while maintaining their health (57). In Europe, there are countries where SEM is not a medical specialty (150–152). As a result, athletes’ screening programs performed by a pediatrician may differ. Pre-participation physical evaluations certified by SEM are mandatory or strongly recommended mainly for competitive sports (151). In opinion of the EFSMA, it is essential that medical specialists involved in sport and exercise medicine aspire to create a standard of care for athletes at all levels, including a standardized digital PPE among European countries (29, 58).

## Summary

Currently, there are no uniform and accepted guidelines for a PPE protocol to be implemented by primary care physicians in Europe for the general pediatric population. The first part of the EAP and the ECPCP position statement presents and summarizes the criteria which are considered to be the most frequently practiced in sports PPE based on expert opinion, reflecting an analysis of the current literature. The aim of this statement is to assist health professionals in implementing the best management programs for sports PPE in individual children and adolescents.

In the absence of a uniform protocol, it is necessary to respect physician autonomy for choosing and implementing the most appropriate and familiar PPE screening strategy and to discuss the decisions made with young athletes and their families (117). This first part of the Position Statement concerning Sport Activities for Children and Adolescents is dedicated to healthy young athletes and does not cover all problems of sport participation in the pediatric population.

## EAP and ECPCP statement for pediatrician and primary care pediatricians concerning pre-participation physical evaluation in young athletes: summary

1. The utility of the sports PPE in the healthy population has been questioned in recent years, but the EAP and the ECPCP strongly recommend performing such evaluation in children and adolescents because the main scope of sports PPE is to maximize safe participation in the pediatric population of athletes.
2. In European countries, local and state requirements determine who is authorized to carry out sports PPE: usually these are general practitioners or pediatricians, but in some countries, the determination of sports eligibility depends on the opinion of the sport and exercise medicine specialist, especially for top-level competitive sports.
3. The timing of the sports PPE should be several weeks before a competition in order to incorporate additional examinations or treatment if needed and should be repeated at least every 1–2 years or more frequently depending on government, state, or sport society requirements, in selected type of sports or for selective health problems in athletes, especially in top-level athletes.
4. In the opinion of the EAP and the ECPCP, sports PPE should include diagnostic elements such as health status, anthropometry, functional capacity, and exercise capacity. The PPE history is designed to identify athletes at risk, especially in athletes with a family history of sudden death. Parental verification of individual and family history is recommended. A physical examination should include an evaluation of biological maturation and vital signs such as visual acuity and a cardiovascular, pulmonary, abdominal, skin, genitalia, neurologic, and musculoskeletal assessment.
5. The EAP and the ECPCP recommend educating athletes during the PPE in topics such as injury prevention or concussion, first-aid and external defibrillator–cardioverter support performance, dietary counseling, the effects of nutritional supplements, and doping practices.
6. Because most of the cases of cardiac death in pediatric athletes occur during exercise in individuals with pre-existing cardiac abnormalities, mostly due to inherited or congenital cardiac disorders, the EAP and the ECPCP strongly recommend to apply protocol proposed by the ESC for a cardiovascular PPE of athletes for the prevention of SAD; the so-called “the Lausanne recommendations.”
7. In the opinion of the EAP and the ECPCP, an electrocardiogram is a very sensitive screening tool for young athletes to determine heart disease or cardiomyopathies. According to ESC recommendations, it is recommended to include an ECG in a PPE cardiovascular screening before practicing sports.
8. Healthcare providers should recognize the individual risks of a pediatric athlete participating in a chosen sport discipline because some sport disciplines may have particular constraints and are subject to a specific medical assessment.
9. Young athletes should be classified as (1) medically eligible for sports performance without restriction, (2) medically eligible with recommendation for further evaluation, or (3) under treatment or not medically eligible for sports performance for all sport disciplines or a selected discipline temporary or constantly contraindicated. Young athletes should receive recommendations regarding appropriate levels of PA and exercise.
10. The EAP and the ECPCP encourages collaborative decision-making with the athlete and his or her family or caregiver.
11. Serious consideration needs to be given to return-to-play policies regarding COVID-19 infections in the pediatric and adolescent population. The EAP and the ECPCP strongly recommend that a history of COVID-19 infection and symptoms also be included in the PPE, and any young athlete who tests positive for current COVID-19 infection, even if asymptomatic, should cease all trainings and games until medical approval is obtained.
12. The aim of presented first part of the EAP and the ECPCP position statement is to assist healthcare professionals in implementing the best management strategies for sports pre-participation in individual children and adolescents.
13. In the absence of a uniform sports PPE protocol for children and adolescents in Europe, this EAP and ECPCP statement is not a formal recommendation for PPE, but rather a summary of practical applications and suggestions based on a current narrative review. In the opinion of the EAP and the ECPCP, it is necessary to respect physician autonomy for choosing and implementing the most appropriate and familiar PPE screening strategy and to discuss the decisions made with young athletes and their families.
